# The effectiveness of specialist roles in mental health metabolic monitoring: a retrospective cross-sectional comparison study

**DOI:** 10.1186/s12888-014-0234-7

**Published:** 2014-09-02

**Authors:** Brian McKenna, Trentham Furness, Elizabeth Wallace, Brenda Happell, Robert Stanton, Chris Platania-Phung, Karen-leigh Edward, David Castle

**Affiliations:** NorthWestern Mental Health, The Royal Melbourne Hospital, Level 1 North, City Campus, Grattan Street, Parkville, Victoria 3050 Australia; School of Nursing, Midwifery and Paramedicine, Australian Catholic University, 115 Victoria Parade, Fitzroy, Victoria 3065 Australia; Institute for Health and Social Science Research Centre for Mental Health Nursing Innovation, School of Nursing and Midwifery, CQUniversity, Bruce Highway, Rockhampton, Queensland 4702 Australia; St. Vincent’s Private Hospital Melbourne Nursing Research Unit, St. Vincent’s Private Hospital Melbourne, 59-61 Victoria Parade, Fitzroy, Victoria 3065 Australia; Faculty of Health Sciences, Australian Catholic University, 115 Victoria Parade, Fitzroy, Victoria 3065 Australia; St. Vincent’s Hospital Mental Health, St. Vincent’s Private Hospital Melbourne, 59-61 Victoria Parade, Fitzroy, Victoria 3065 Australia; Department of Psychiatry, University of Melbourne, Grattan Street, Parkville, Victoria 3050 Australia

**Keywords:** Mental health, Metabolic monitoring, Nursing, Cardiovascular diseases

## Abstract

**Background:**

People with serious mental illness (SMI) exhibit a high prevalence of cardiovascular diseases. Mental health services have a responsibility to address poor physical health in their consumers. One way of doing this is to conduct metabolic monitoring (MM) of risk factors for cardiovascular diseases. This study compares two models of MM among consumers with SMI and describes referral pathways for those at high risk of cardiovascular diseases.

**Methods:**

A retrospective cross-sectional comparison design was used. The two models were: (1) MM integrated with case managers, and (2) MM integrated with case managers and specialist roles. Retrospective data were collected for all new episodes at two community mental health services (CMHS) over a 12-month period (September 2012 – August 2013).

**Results:**

A total of 432 consumers with SMI across the two community mental health services were included in the analysis. At the service with the specialist roles, MM was undertaken for 78% of all new episode consumers, compared with 3% at the mental health service with case managers undertaking the role. Incomplete MM was systemic to both CMHS, although all consumers identified with high risk of cardiovascular diseases were referred to a general practitioner or other community based health services. The specialist roles enabled more varied referral options.

**Conclusions:**

The results of this study support incorporating specialist roles over case manager only roles for more effective MM among new episode consumers with SMI.

## Background

People with serious mental illness (SMI: psychotic illnesses primarily schizophrenia spectrum or bipolar affective disorder) have a reduced life expectancy compared with the general population [1]. People with SMI exhibit a substantially higher prevalence of co-existing conditions such as cardiovascular diseases [1] and its precursor, metabolic syndrome [2,3]. Such is the sub-optimal combination of mental and physical health that the risk of cardiovascular diseases alone, is between two [2] and 12 times greater among people with SMI than the general population [4]. Australian mental health services have a responsibility to address poor physical health [5], and this will involve a shift in provider culture, practice, and service delivery [6].

A necessary process in the shift of provider practice is to conduct metabolic monitoring (MM). Such monitoring involves quantifying risk factors for cardiovascular diseases such as blood pressure, cholesterol, blood sugar levels, waist and hip circumference, and body mass and stature [6,7], along with modifiable lifestyle behaviours (e.g., tobacco smoking, alcohol consumption, physical activity, and nutrition) and antipsychotic medication prescription. However, selecting and implementing a MM model can be problematic due to perceptions of efficacy within services and staff [6,8–10]. Furthermore, pessimism persists about the effect of healthy lifestyle recommendations post MM on consumer behaviours [1] and health outcomes [11].

The principle aim of effective MM of people with SMI is to obtain metabolic information that can be used to tailor physical health interventions [12]. However, a generic MM model may be challenged by the aforementioned reasons [12]. Furthermore, effectiveness of MM has not been described with randomised controlled trials at this time [12]. Therefore, clinicians are without a clear MM model and confusion among who is responsible for MM (i.e., primary or secondary care) persists [13,14].

Despite evidence supporting the need for MM of people with SMI and the development of clinical guidelines for the physical health care of consumers [15–17], the process of monitoring is often incomplete [6,18,19]. Incomplete monitoring implies poor identification of cardiovascular diseases risk factors [20,21] and subsequent increased morbidity [17]. Furthermore, adequate and responsible referral once risk has been identified should also supplement MM as a fundamental component in addressing identified risks [22,23], cognisant of the reported communication barriers among mental health and community health services [24].

Given the known risk of people with SMI to cardiovascular diseases, more knowledge is needed about the effectiveness of MM models and associated referral pathways for high risk consumers to inform future physical health policy across mental health services. Therefore, the aims of this study were to; (1) describe the effectiveness of MM and scope of referral pathways within one of Australia’s largest mental health services, and (2) compare MM in an area with specialist MM roles with an area reliant of existing case manager roles.

## Methods

### Setting

Responding to the challenges of effective MM, one of Australia’s largest mental health services introduced a MM policy to improve the early detection, treatment, and use of referral pathways among consumers with SMI. The policy initiative was coupled with staff education that occurred across all community mental health services (CMHS) in 2011–12. Metabolic monitoring was supported with the provision of a generic MM data collection form and a risk identification/referral pathway algorithm. The identification of risk was based on national (e.g., Heart Foundation [25]) and international (e.g., World Health Organization [26]) guidelines. As such, the responsibility for MM and associated referral pathways was integrated into existing case manager roles for all CMHS. As per the MM policy, medical staff and case managers have the shared responsibility to ensure that each consumer has had MM. Furthermore, case managers should support consumers to access appropriate investigations and treatments including facilitating attendance to general practitioners or other medical appointments. In the instance of detected high risk of cardiovascular diseases, the case manager should facilitate referral to and attendance at the consumer’s general practitioner. The procedure for collection of MM information in line with the service policy is for case managers to collect data (e.g., body mass and blood pressure), which is recorded on the generic MM data collection form. Case managers are required to facilitate medical investigations such as collection of bloods for fats and sugar investigations.

In addition, one CMHS saw value in developing specialist roles within existing mental health nursing staff resource to support case managers undertaking MM and physical health assessment of consumers. The equivalent of 0.5 EFT of Registered Nurse and 0.8 EFT Enrolled Nurse was allocated to focus on supporting case managers undertake MM and to ensure the form has been completed and missing information is updated.

### Design

A retrospective cross-sectional comparison study was undertaken to address the research aims. Two CMHS within the mental health service were selected for comparison; (1) a CMHS with the specialist MM roles and (2) a CMHS with the responsibility for MM resting with case managers. The populations of both CMHS catchment areas are characterised with low socio-economic status and high immigrant and ethnic diversity compared with the remainder of the state of Victoria. All new consumer episodes referred to each CMHS for the first time, inclusive of September 2012 to August 2013 were assessed for MM data. A retrospective electronic file audit was undertaken and data were collected based on the presence of the aforementioned generic MM data collection form. The absence of the form was indicative of MM not having been undertaken. From the form, the presence of monitoring specific risk factors of cardiovascular diseases was determined. The MM variables were body mass index, fasting blood glucose, blood pressure, waist circumference, total cholesterol, triglycerides, low density lipoprotein level, high density lipoprotein level, exercise status, and smoking status. Metabolic monitoring outcome variables were categorised for cardiovascular diseases risk as ‘high risk’, ‘moderate risk’, and ‘low risk’ in accordance with national and international guidelines. The scope of referral pathways were described relative to primary health and preventative health providers. The conduct of the study was ethically approved by the Melbourne Health Office for Research (QA2013174).

### Statistical analyses

Descriptive statistics were computed for consumer demographics, MM variables, and referral pathways. A univariate Chi-square (*χ*^*2*^) analysis was conducted to compare the difference among frequency counts of the generic MM data collection form across the two CMHS with Statistical Package for Social Scientists 20.0 for Windows (IBM Corp, Armonk, USA). Significance was accepted at *p* ≤ 0.05.

## Results

A total of 432 new episode consumers were eligible for MM across the two CMHS (see Table 1). The samples were evenly matched according to gender. Consumer age range was 19 years to 65 years. Most consumers were diagnosed with a psychotic illness (schizophrenia, schizoaffective disorder, or psychotic illness unspecified).

**Table 1:** **Sample descriptors (N = 432) across the two CMHS**

		**SR** ^**1**^ **CMHS**	**CM** ^**2**^ **CMHS**
		**(** ***n*** **= 134)**	**(** ***n*** **= 298)**
Gender (%)	Male	67 (50)	159 (53)
	Female	67 (50)	139 (47)
Age (years)	Male mean (SD)	38 (11)	38 (10)
	Range	19-63	19-64
	Female mean (SD)	38 (11)	41 (12)
	Range	18-64	20-65
Diagnosis (%)	Psychotic illness^3^	84 (63)	188 (63)
	Bipolar affective disorder unspecified	18 (12)	22 (7)
	Major depression	3 (2)	17 (6)
	Other diagnosis^4^	31 (23)	71 (24)

NOTE: SR CMHS case manager EFT = 10.6. CM CMHS case manager EFT = 17.4.

^1^Specialist roles.

^2^Case manager only roles.

^3^Schizophrenia, schizoaffective disorder, psychotic disorder unspecified.

^4^Other ICD-10-AM ‘F’ codes.

At the CMHS with specialist roles, MM was undertaken for 105 (78%) of 134 new episode consumers. At the CMHS with case manager only roles, MM was undertaken for 8 (3%) of the 298 new episodes, *χ*^2^(1, N = 113) = 83.27, *p* = 0.01.

At the CMHS with the specialist MM roles smoking status, exercise frequency, low density lipoprotein level, and waist circumference were the most common ‘high risk’ variables for cardiovascular diseases (see Table 2). Of new episode consumers that were metabolically monitored at the specialist roles CMHS, the range of monitoring of outcome variables was 53% (low density lipoprotein) to 100% (smoking status). At the CMHS with case managers responsible for undertaking MM, a lack of effectiveness of MM was found.

**Table 2:** **Metabolic monitoring risk outcomes of new episode consumers across the two CMHS**

**Metabolic monitoring outcome variables at the CMHS with specialist roles**	**Cardiovascular diseases risk factors**			
**(** ***n*** **= 105)**	***n*** **(%)**			
	**High**	**Moderate**	**Low**	**Missing**
Body mass index^1^	21 (30)	22 (31)	27 (39)	35
Fasting blood glucose^2^	5 (8)	12 (20)	44 (72)	44
Blood pressure^3^	2 (3)	4 (6)	59 (91)	40
Waist circumference^4^	33 (51)	16 (24.5)	16 (24.5)	40
Total cholesterol^5^	24 (36)	-	42 (64)	39
Triglycerides^6^	23 (35)	-	43 (65)	39
Low density lipoproteins^7^	37 (66)	-	19 (34)	49
High density lipoproteins^8^	5 (9)	-	52 (96)	48
Exercise status^9^	44 (76)	-	14 (24)	47
Smoking status^10^	34 (32)	-	71 (68)	0
**Metabolic monitoring outcome variables at the CMHS with case manager only roles**	**Cardiovascular diseases risk factors**			
**(** ***n*** **= 8)**	***n*** ^**11**^			
	**High**	**Moderate**	**Low**	**Missing**
Body mass index	2	2	3	1
Fasting blood glucose	0	1	4	3
Blood pressure	0	2	5	3
Waist circumference	3	1	0	4
Total cholesterol	3	-	1	4
Triglycerides	0	-	2	6
Low density lipoproteins	2	-	2	4
High density lipoproteins	1	-	2	5
Exercise status	4	-	1	3
Smoking status	7	-	0	1

^1^High > 30 kg.m^−2^, Moderate 25–30 kg.m^−2^, Low < 25 kg.m^−2^.

^2^High 7.0 mmol.L^−1^, Moderate 5.5-6.9 mmol.L^−1^, Low < 5.5 mmol.L^−1^.

^3^High > 180 mm.Hg^−1^ systolic and > 110 mm.Hg^−1^ diastolic, Moderate 140-180 mm.Hg^−1^ systolic and 90–110 mm.Hg^−1^, Low < 140 mm.Hg^−1^ systolic and < 90 mm.Hg^−1^.

^4^High female ≥ 88 cm, male ≥ 102 cm, Moderate female ≥ 80 cm, male ≥ 94 cm, Low female < 80 cm, male < 94 cm.

^5^High > 5.5 mmol.L^−1^, Low ≤ 5.5 mmol.L^−1^.

^6^High > 2.0 mmol.L^−1^, Low ≤ 2.0 mmol.L^−1^.

^7^High > 2.5 mmol.L^−1^, Low ≤ 2.5 mmol.L^−1^.

^8^High < 1.0 mmol.L^−1^, Low ≥ 1.0 mmol.L^−1^.

^9^High < every day, Low = every day.

^10^High = current smoker, Low = does not smoke.

^11^Percentage not reported for the case manager only roles CMHS due to low *n*.

Of new episode consumers who underwent MM (n = 113), referral pathways were established for each identified as ‘high risk’ of cardiovascular diseases across both CMHS. Referral pathways at the specialist roles CMHS included; a dietician, an exercise physiologist, a well women’s group, a diabetic educator, a podiatrist, council initiatives (Jamie's Ministry of Food and a leisure programme), a YMCA exercise programme, a Quit programme for smoking cessation, a volunteer fitness instructor, and general practitioners. The referral pathway at the CMHS with case manager only roles was to each consumer’s general practitioner.

## Discussion

The results of this study underline the importance of specialist roles to more effectively support MM in CMHS. In this study, specialist roles were able to document varying levels of cardiovascular diseases risk among 78% of new episode consumers compared with 3% at the CMHS where MM was a usual work task of case managers. These results support the opinions of many clinicians; that an individual should be tasked with MM within mental health services and that extra discrete resourcing is required [27–29]. However, the specialist roles described in the current study were created by reconfiguring existing mental health nursing roles rather than a requirement of additional resource incurred by the CMHS.

Despite specialist roles in the current study, smoking status was the only outcome variable with complete monitoring among all 113 new episode consumers with SMI. Gaps were found in the reporting of specific risk factors, despite the gains made in undertaking MM with the total caseload. These findings support literature describing the absence or neglect of blood pressure [24], waist circumference, and body mass monitoring [6]. Retrospective informal discussion with the specialist role mental health nurses revealed several factors limiting thorough and systemic MM at the CMHS; poor adherence to blood profiling attributed to consumer anxiety towards needles, procedural complexity in collecting fasting blood glucose, the logistical barriers to attending community based pathology services, and poor communication between the mental health service and local medical officers regarding the transfer of diagnostic results. Similar opinions have been expressed and reported in previous research [28,30–32]. Acuity among some consumers and refusal to consent to participate in MM were also discussed and are commonly identified as potential barriers to MM [33].

Additional barriers to completing MM have included across-discipline confusion about who should be responsible for the role [6,11,28] and a lack of supporting procedures [23]. Furthermore, MM has also been limited by exclusion of the supportive capacity of carers [1]. Such barriers may explain the poor MM at the CMHS in this study, where the responsibility of monitoring relied on case managers completing the process as specified in the service policy. Regardless of the consumer and service barriers, the volume of missing data among consumers with SMI in the current study is alarming considering the number of consumers identified at high risk of cardiovascular diseases.

The scope of referral pathways for high risk consumers in this study provided evidence of collaboration with primary health and preventative health providers. For the CMHS without specialist roles, referral followed service policy (i.e., to the consumer’s general practitioner). For the CMHS with specialist roles, referral pathways were tailored to the MM risk factor identified. These results support the importance of tailored referral pathways established by a specialist nurse role in the United Kingdom [23]. Referrals to physical activity and body mass management groups [23,34] and one-to-one tailored health interventions [35] have been effective in eliciting positive lifestyle changes among consumers with SMI.

Future research should focus on mental health services developing strategies to engage consumers with MM, so that all recommended cardiovascular diseases risks are disclosed. Furthermore, given the emerging knowledge of preferences for interventions among consumers with SMI [34,35], guidance is also required about efficacy of interventions to reduce cardiovascular diseases risk once MM is systemic to mental health services.

### Limitations

This study was delimited to the comparison of two CMHS within one of Australia’s largest mental health services. A more thorough understanding of the effectiveness of the MM policy within the mental health service could be described if all CMHS were retrospectively audited. Furthermore, we are only able to anecdotally speculate why MM did not occur for 22% of consumers at the specialist roles CMHS and 97% of consumers at the case manager roles CMHS. This study focused on two aims; (1) effectiveness of two MM models and (2) referral pathways of consumers identified at high risk of cardiovascular diseases. Given that the specialist roles occurred within existing budget and required a re-configuration of existing mental health nursing roles, there is no indication of the impact of the re-configuration on overall service delivery.

## Conclusions

The results of this study support CMHS specialist roles over case manager only roles for more effective MM among new episode consumers with SMI. The specialist roles were also able to provide consumers with more tailored referral options.

## Abbreviations

SMI: Serious mental illness
MM: Metabolic monitoring
CMHS: Community mental health service
EFT: Equivalent full time
